# IL-15: from discovery to FDA approval

**DOI:** 10.1186/s13045-025-01664-8

**Published:** 2025-02-18

**Authors:** Zihai Li, John Wrangle, Kai He, Jonathan Sprent, Mark P. Rubinstein

**Affiliations:** 1 Pelotonia Institute for Immuno-Oncology, The Ohio State University, Columbus, OH 43210 USA; 2 Division of Medical Oncology, Department of Internal Medicine, College of Medicine, The Ohio State University, Columbus, OH 43210 USA; 3 Department of Microbial Infection and Immunity, The Ohio State University, Columbus, OH 43210 USA; 4 Division of Hematology and Oncology, Department of Medicine, Medical University of South Carolina, Charleston, SC 29425 USA; 5 Department of Microbiology and Immunology, Medical University of South Carolina, Charleston, SC 29425 USA; 6 Immunology Division, Garvan Institute of Medical Research, Darlinghurst, 2010 Australia; 7 St. Vincent’s Clinical School, University of New South Wales, Sydney, 1466 Australia

In April 2024, the FDA approved the interleukin (IL)−15 superagonist, N-803 (Anktiva, nogapendekin alfa inbakicept-pmln), for the treatment of bladder cancer [1]. This is the first cytokine in over 30 years to receive FDA approval for the treatment of cancer, and the culmination of years of preclinical and clinical studies involving both academic- and industry- driven research.

To understand the steps leading to this landmark approval, it is helpful to review some key historical events (Fig. 1). Notably, the first cytokines FDA approved for the treatment of cancer were interferon (type 1) (1986, hairy cell leukemia) and IL-2 (1992, renal cell carcinoma) [2]. Within a few years of their initial approvals, both cytokines would also receive other FDA approvals including for the treatment of metastatic melanoma. While both cytokines have broad immune stimulatory activities, IL-2 is unique in that it is also a powerful lymphocyte growth factor [3–6]. These qualities led to the evaluation and use of IL-2 with many other experimental immunotherapies including adoptive cell therapy. Notably, the adoptive transfer of tumor infiltrating lymphocytes (TIL) in combination with IL-2 first showed efficacy in human patients in the late 1980s [7]. After decades of work, in February 2024, TIL (lifileucel) in combination with IL-2 received FDA approval for the treatment of melanoma [8], which is the first approved adoptive cell therapy using lymphocytes for the treatment of a solid tumor.

**Fig. 1:**
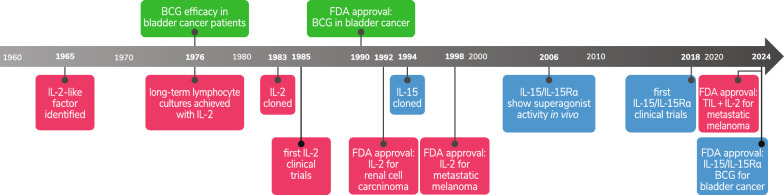
Timeline of key events related to the discovery and development of IL-15 and BCG as therapeutics

Despite its established efficacy, IL-2 has a short half-life, and at approved doses, IL-2 can induce life threatening toxicities [9]. This side effect profile likely severely curtailed subsequent clinical development. Thus, high dose IL-2 as a monotherapy was FDA approved for renal cell carcinoma and metastatic melanoma, clinical development for other indications halted despite evidence of efficacy in other cancers [10]. Thus began the effort to develop alternatives with a similar mechanism of action.

The discovery of IL-15 in 1994 was the first step in the development of a promising alternative to IL-2 [4–6, 11–13]. Like IL-2, IL-15 is a powerful lymphocyte growth factor for CD8^+^ T cells and NK cells. Both IL-2 and IL-15 induce similar intracellular signaling through the shared IL-2 receptor (R) βγ subunits. However, while the private IL-2Rα (CD25) subunit improves responsiveness to limiting amounts of IL-2, IL-15 signals independently of IL-2Rα. Because IL-2Rα is expressed at high and constitutive levels on immune suppressive T regulatory cells, an advantage of IL-15 is that it does not expand T regulatory cells. By itself, IL-15 can induce anti-tumor activity in preclinical models [4, 5, 13]. Interestingly, IL-15 can be induced by type I interferon signaling [14, 15], which suggests a mechanism for type 1 interferon-mediated anti-tumor efficacy. Further differentiating IL-15 from IL-2 is the private IL-15Rα subunit. While thought to allow for high affinity cytokine binding, the description of IL-15Rα as a receptor is somewhat of a misnomer as IL-15Rα can transpresent IL-15 either on the cell surface or in soluble format [4, 16]. Building off this knowledge, we and others found that pre-association of soluble IL-15Rα subunit with IL-15 (with or without an Fc) could dramatically improve biological activity and half-life of IL-15 in vitro and in vivo, and these IL-15/IL-15Rα complexes had potent anti-tumor activity [17–21]. Based on this concept (Fig. 2), several companies have developed clinical grade reagents including N-803 (Anktiva), NIZ985, SOT101 (Nanrilkefusp alfa), and XmAb24306 (Efbalropendekin alfa) [22–26].

**Fig. 2:**
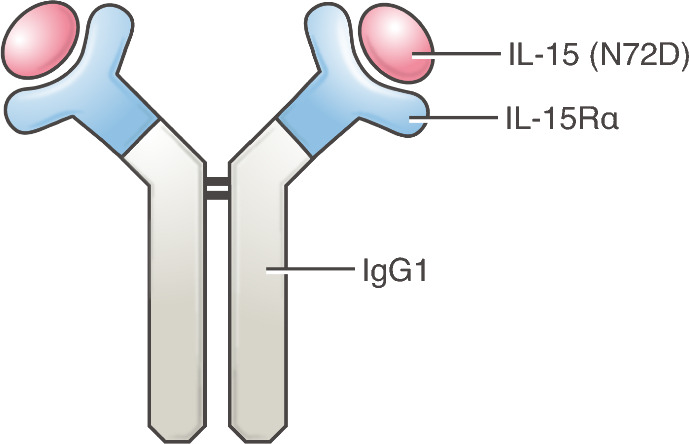
Diagram of N-803 (IL-15/IL-15Rα cytokine complexes) including IL-15, IL-15Rα, and the Fc region from IgG1

These IL-15 superagonists have now been evaluated in a wide range of clinical trials including patients with cancer and HIV-1 infection [23–30]. Similar to animal studies, human trials have shown IL-15/IL-15Rα complexes have improved half-life and cytokine-induced biological activity on human lymphocytes including CD8^+^ T cells and NK cells when compared to free IL-15 [25, 31, 32]. Importantly, at the doses used, this biological activity was obtained without the severe toxicities or need for in-hospital administration associated with high dose IL-2. For the agent in most advanced clinical testing, N-803, single agent activity was shown in patients with hematological malignancies who relapsed after allogeneic hematopoietic cell transplantation [25]. We and others have shown these agents can be administered safely in combination with other immunotherapy agents and with encouraging efficacy signals [26, 28, 30]. Thus, the combination of N-803 and anti-PD-1 mAb (nivolumab) could be given safely to NSCLC patients [30], and there is an ongoing phase III trial (NCT03520686). Taking advantage of the expansion and activation of Fc receptor-expressing immune cells such as NK cells that can mediate enhanced antibody-dependent cellular cytotoxicity, IL-15-based agents show the ability to enhance the efficacy of tumor-targeting antibodies [33]. Clinically, success with this concept has been demonstrated by combination of N-803 with rituximab (anti-CD20 mAb) [28]. Another appealing approach, not yet assessed clinically, is the use of systemic IL-15 agents instead of high dose IL-2 to support adoptively transferred T cells, such as TIL, administered after lymphodepletion. In addition to approaches using early generation IL-15 agents, it is notable that other approaches are in development including the use of fusion proteins combining IL-15 with targeting moieties (such as directing IL-15 to PD-1 expressing lymphocytes) and genetically encoded membrane-bound IL-15 [34, 35]. Expression of membrane-bound IL-15 on adoptively transferred lymphocytes may improve functional capacity and reduce the need for lymphodepleting chemotherapy [36].

The treatment of non-muscle invasive bladder cancer (NMIBC) involves the use of intravesical bacillus Calmette-Guerin (BCG) to avoid cystectomy [37, 38]. BCG was initially developed as a vaccine for tuberculosis, and since initial human testing in 1921, has been the most widely used vaccine worldwide [37–39]. Interest in using BCG for cancer therapy resulted from work by William Coley and others showing that bacterial agents may trigger anti-tumor immunity [40, 41], and BCG use in cancer patients was reported as early as 1936 [42]. Direct evidence in support of the use of BCG to induce anti-tumor immunity was supported by animal studies conducted by Lloyd Old, Burton Zbar, and others [43–47]. Evidence of BCG-mediated anti-tumor activity in humans was reported as early as 1965 in a mixed patient population [48]. Soon thereafter, evidence of BCG-mediated anti-tumor activity was reported in acute lymphoblastic leukemia [49], metastatic melanoma [50], and other cancers [37, 38, 51]. While our interpretation of the use of BCG as a cancer immunotherapy might be altered with today’s modern understanding of immunology, the practical clinical results of BCG therapy in bladder cancer would prove most impactful. Thus, in 1976 Alvaro Morales reported favorable responses in 9 patients with superficial bladder cancer treated as part of the first human trial using intravesical BCG [52]. This work was followed by randomized trials confirming efficacy [53–56], leading the FDA to approve BCG for NMIBC in 1990 [37].

While intravesical BCG can lead to durable responses in NMIBC, up to 40% of patients have disease recurrence at which time patients are thought to be unresponsive to additional BCG administration [54, 57, 58]. With the goal of improving BCG-mediated anti-tumor immunity, preclinical bladder cancer studies in rats demonstrated the combination of IL-15 and BCG delivery by intravesical instillation was safe and improved anti-tumor immunity [59–61]. The mechanism of action in these preclinical studies was not fully established, but responses were associated with expansion of NK cells and inflammatory cytokines. Given the encouraging preclinical studies, the combination of intravesical N-803 and BCG was evaluated in NMIBC patients, and in a phase I trial, encouraging safety, tolerability, and long-term outcomes were observed [62]. Intravesical N-803 given with BCG was then evaluated in a single-arm, multicenter trial of BCG-unresponsive, high-risk NMIBC patients with carcinoma in situ with or without papillary tumors [63] (NCT0302285, QUILT-3.032). In data reported by the FDA, 62% of the 77 patients enrolled had a complete response, with 58% of patients sustaining this complete response for 12 months. This response rate far exceeded guidance of 30% complete response at 1 year set by FDA experts and the International Bladder Cancer Group [64–66]. As a result, in April 2024 the FDA approved N-803 with BCG for this patient population [1], which represents the first FDA approval of any IL-15 based therapy.

While this FDA approval represents a promising step for patients, critical questions remain including the need for in-depth studies to establish the mechanism of action of the combination of N-803 and BCG. The latter will be helpful for the design of future therapies for bladder cancer patients. More broadly, this FDA approval is noteworthy as it represents the first of a new era of approvals for cytokines in the treatment of cancer. For IL-15 superagonists, future approvals might be for recombinant protein or genetically encoded constructs, however, it may also be for an IL-15-concept not yet envisioned.

## Data Availability

No datasets were generated or analysed during the current study.
